# The National Integrated Project for Prospective Observation of Non-communicable Disease and its Trends in the Aged 2010 (NIPPON DATA2010): Objectives, Design, and Population Characteristics

**DOI:** 10.2188/jea.JE20170240

**Published:** 2018-03-05

**Authors:** Aya Kadota, Nagako Okuda, Takayoshi Ohkubo, Tomonori Okamura, Nobuo Nishi, Hirotsugu Ueshima, Akira Okayama, Katsuyuki Miura

**Affiliations:** 1 Center for Epidemiologic Research in Asia, Shiga University of Medical Science, Shiga, Japan; 2 Department of Public Health, Shiga University of Medical Science, Shiga, Japan; 3 Department of Health and Nutrition, University of Human Arts and Sciences, Saitama, Japan; 4 Department of Hygiene and Public Health, Teikyo University School of Medicine, Tokyo, Japan; 5 Department of Preventive Medicine and Public Health, Keio University School of Medicine, Tokyo, Japan; 6 International Center for Nutrition and Information, National Institute of Health and Nutrition, National Institutes of Biomedical Innovation, Health and Nutrition, Tokyo, Japan; 7 Research Institute of Strategy for Prevention, Tokyo, Japan

**Keywords:** study profiles, NIPPON DATA2010, cohort study, cardiovascular diseases, Japanese

## Abstract

**Background:**

The structure and risk factors for cardiovascular diseases (CVD) in Japan may change because lifestyle, particularly nutrition, socioeconomic status, and medical care, which affect CVD, may markedly change over time. Therefore, a new prospective cohort study on a representative general Japanese population based on national surveys is required.

**Methods:**

In November 2010, the baseline survey of the National Integrated Project for Prospective Observation of Non-communicable Disease and its Trends in the Aged 2010 (NIPPON DATA2010) was performed with the National Health and Nutrition Survey of Japan (NHNS2010) in 300 randomly selected districts throughout Japan. The survey included a questionnaire, electrocardiogram, urinalysis, and blood biomarkers added to the NHNS2010 examinations. Physical measurements, blood biomarkers, and dietary data were also obtained in NHNS2010. Socioeconomic factors were obtained by merging with the Comprehensive Survey of Living Conditions 2010 (CSLC2010) dataset. Participants are followed annually for the incidence of diabetes mellitus, CVD events (acute coronary events, heart failure, atrial fibrillation, and stroke), and cause-specific mortality. The activities of daily living are followed every 5 years.

**Results:**

A total of 2,898 individuals aged 20 years or older agreed to participate in the baseline survey of NIPPON DATA2010. The participation rate was 74.6%. Of these, data from NHNS2010 was merged for 2,891 participants (1,236 men and 1,655 women). The data of 2,807 participants were also merged with CSLC2010 data.

**Conclusions:**

We established NIPPON DATA2010 as a cohort study on a representative general Japanese population that covers all of Japan.

## BACKGROUND AND PURPOSE

Cardiovascular disease (CVD) is the leading cause of death worldwide and adversely affects healthy life expectancy.^1^^,^^2^ The National Survey of Circulatory Disorders (NSCD) has been conducted in Japan every decade since the 1960s by the Ministry of Health and Welfare in order to assess the current status of CVD among Japanese adults for the development of future preventive measures.^3^ The study group of the National Integrated Project for Prospective Observation of Non-communicable Disease and its Trends in the Aged (NIPPON DATA) conducted long-term cohort studies on participants of the 3^rd^ NSCD in 1980 and 4^th^ NSCD in 1990, when the Japanese economy was growing, named NIPPON DATA80 and NIPPON DATA90.^4^^–^^8^ Many findings from our studies were utilized in preventive measures, such as the National Health Promotion Movement in the Twenty-first Century (Health Japan 21) and Health Japan 21 (the second term).^9^^,^^10^

The structure of diseases in Japan may have subsequently changed because of the rapid aging of its population, together with changes in lifestyle, medical care, and socioeconomic status (SES) based on the low-growth economy.^11^^,^^12^ Under these conditions, the expansion of health inequality has been reported.^13^ Although the social determinants of health inequality need to be identified in order to prompt political action, the effects of SES on health have not yet been fully investigated in Japan.^14^^,^^15^ Therefore, a new cohort study on a representative general Japanese population based on national surveys that cover all of Japan with standardized methods and enable assessments of health-related factors from multiple aspects, including SES, is required. We herein describe the objectives, design, and characteristics of a new cohort study, NIPPON DATA2010, which started in 2010 with the purpose of monitoring and revealing factors related to CVD in a recent Japanese population.

## METHODS

### Study participants

**Figure 1.**
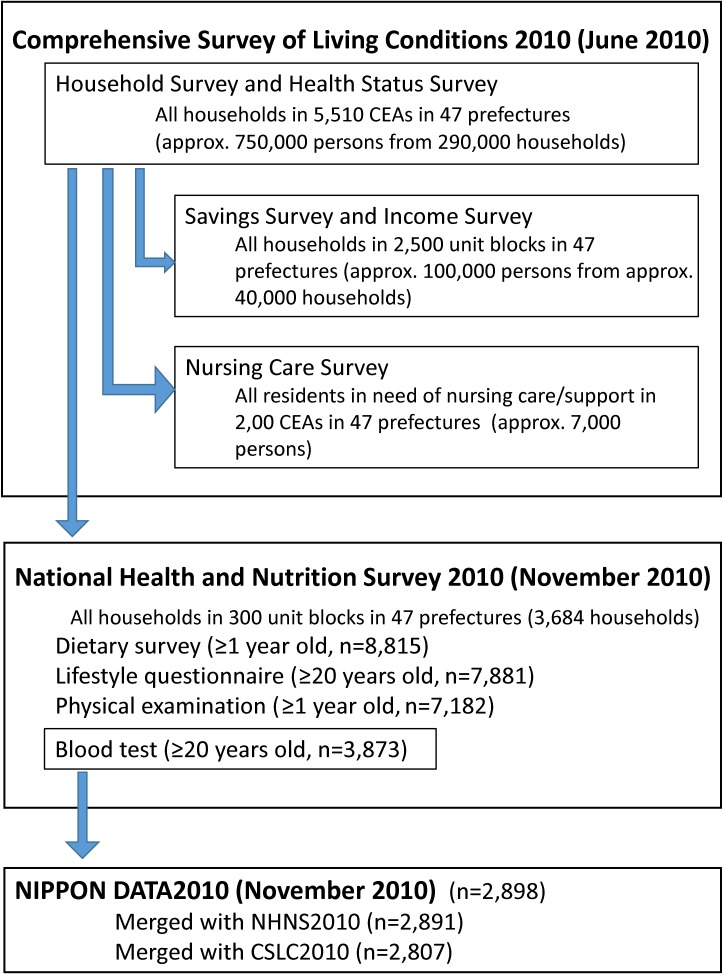
Study population of NIPPON DATA2010. The 5,510 CEAs selected for CSLC2010 were divided into 11,000 unit blocks consisting of 20–30 households each for NHNS2010 sampling. CEA, census enumeration area; CSLC2010, Comprehensive Survey of Living Conditions 2010; NHNS2010, National Health and Nutrition Survey 2010.

NIPPON DATA2010 was established as a prospective cohort study of participants of the National Health and Nutrition Survey of Japan in 2010 (NHNS2010) and Comprehensive Survey of Living Conditions in 2010 (CSLC2010), which were conducted by the Ministry of Health, Labour and Welfare of Japan (Figure 1).

### CSLC2010

In June 2010, CSLC was conducted in order to survey national living conditions, such as health, medical care, welfare, pension, income, and related factors.^16^ Sampling was based on the national population census enumeration area (CEA), which covered all of Japan. Each CEA consisted of approximately 50 households. In the 2010 survey, approximately 290,000 households from 5,510 randomly selected CEAs were sampled for the Household Survey and Health Status Survey. The response rate was 79.4%. Among these CEAs, the Saving Survey and Income Survey covered 2,500 randomly selected unit blocks; each unit block consisted of 20–30 households, approximately half of which were in the CEA. The Nursing Care Survey also covered 2,500 unit blocks that partially overlapped. Detailed information on CSLC was described elsewhere.^16^

### NHNS2010

Among the 5,510 CEAs (11,000 unit blocks) for CSLC2010, 300 unit blocks from throughout Japan were randomly selected for NHNS2010. All household members who resided in the blocks and participated in CSLC2010 were announced to participate in NHNS2010 in November 2010. A total of 8,815 residents aged 1 year and older from 3,684 households participated in the survey (Figure 1). The participation rate by households was 68.8%. Adult participants aged 20 years or older were also asked to take lifestyle questionnaires and blood examinations for NHNS2010, in addition to the dietary survey and physical examination, with 7,881 (2,672 men and 4,209 women) taking the lifestyle questionnaire and 3,873 (1,598 men and 2,275 women) taking the blood examination for NHNS2010. Details of NHNS2010 were described elsewhere.^17^^,^^18^

### NIPPON DATA2010

The baseline survey for NIPPON DATA2010 was performed simultaneously with NHNS2010 in November 2010.^19^^,^^20^ Trained staff explained the aim and methods of NIPPON DATA2010 to the 3,873 participants aged 20 years or older who underwent the blood examination for NHNS2010 at the NHNS2010 places such as public health centers. A total of 2,898 participants (1,239 men and 1,659 women; participant rate, 74.6%) agreed to participate in the baseline survey for NIPPON DATA2010. Staff obtained written informed consent from all participants before enrollment. The Institutional Review Board of Shiga University of Medical Science (No. 22-29, 2010) approved this study.

Of the 2,898 participants, 7 were excluded because it was not possible to merge data from NHNS2010. Thus, the remaining 2,891 participants (1,236 men and 1,655 women) provided baseline data for NIPPON DATA2010. In the analysis of socioeconomic factors, 2,807 participants who were also merged to CSLC2010 were listed. Regarding the follow-up survey, 2,732 participants (1,170 men and 1,562 women) agreed to be included in the study.

### Measures

The measures of NIPPON DATA2010 were composed of three parts: NHNS2010, CSLC2010, and specific measures in NIPPON DATA2010. The outline of the integrated dataset, variables, and their original survey are listed in Table 1. Health professionals for NHNS2010 and trained staff for NIPPON DATA2010 collected information on smoking, alcohol consumption, and medical history. Lifestyle-related factors, including knowledge of CVD risk factors, were asked using self-administered questionnaires. Regarding physical activity, the time (hours) spent at each activity level was also asked: heavy activity, moderate activity, slight activity, watching television, other sedentary time, and no activity (sleeping). The interviewer then ensured that the total time added up to 24 hours, and the physical activity index was calculated by multiplying the time and corresponding weighting factor estimated in the Framingham study.^21^ The activities of daily living (ADL) were also asked for five aspects: eating, using the toilet, dressing, bathing or taking a shower, and walking, with answers of “independent” or “need assistance”.^22^ Information on instrumental activities of daily living (IADL), intellectual activities, and social roles was also obtained based on the Tokyo Metropolitan Institute of Gerontology Index of Competence.^23^ Socioeconomic factors, such as household composition and monthly household expenditure, were obtained from CSLC2010 with the permission of the Ministry of Health, Labour and Welfare. Equivalent household expenditure (EHE) was estimated using the following formula: EHE = monthly household expenditure/square root of the number of household members. Information about medical insurance and pension was also obtained from CSLC2010. The dietary intake data of NHNS2010, which was assessed by 1-day semi-weighted household dietary records, were also obtained with the allowance of the Ministry of Health, Labour and Welfare. The detailed procedure for the dietary survey was described elsewhere.^17^^,^^18^

**Table 1.** Outline of the integrated NIPPON DATA2010 dataset, variables, and their original surveys

NIPPON DATA2010 specific data (November 2010)
History and treatment of hypertension, diabetes mellitus, and dyslipidemia
Activity of daily living
Educational attainment
Questions for depressive mood (K6)
Knowledge of symptoms/implications of stroke, CHD, and CVD risk factors.
Normal physical activity level (breakdown of 24 hr by Mets categories)
Menopausal status (for women)
Biomarkers (BNP, high sensitivity CRP, urinary Cre, Na, K, and albumin)
Electrocardiogram reading by Minnesota codes
National Health and Nutrition Survey 2010 (November 2010)
Anthropometric measurements
Biomarkers (blood cell counts and blood biochemistry)
Blood pressure
Lifestyle questionnaire
History of stroke, CHD, and renal disease
Engaged in lifestyle modifications
Dental health habits
Smoking habit (including passive smoking)
Drinking habit
History of CVD risk factors
Results from the dietary survey (one-day semi-weighing dietary record)
Food intake (99 foods, per day)
Nutrient intake (42 nutrients, per day)
Comprehensive Survey of Living Conditions 2010 (June 2010)
Household survey
Household composition
Number of household members, marital status, offspring, multigenerational household
Housing (rented/owned, detached/apartment)
Household expenditure
Medical insurance, public pension
Occupation (type of job, size of company)
Health status survey
Subjective symptoms (43 symptoms)
Medical treatment (39 diseases)
Physical impairments affecting daily life
Subjective sense of well-being
Questions for depressive mood (K6)
Physical check-up
Cancer screening

CHD, coronary heart disease; CVD, cardiovascular disease; BNP, brain natriuretic peptide; CRP, C-reactive protein.

Physical measurements were obtained by trained health professionals. They measured blood pressure in duplicate using a standard mercury sphygmomanometer on the right arm of seated participants after 5 minutes of rest. A 12-lead resting electrocardiogram (ECG) was also recorded. Each ECG record was manually read according to the Minnesota codes by two trained researchers independently.^24^^,^^25^ When the coding results mismatched, the central committee of ECG reading adjudicated the codes.

In the baseline survey, casual blood samples were obtained. Serum was separated and centrifuged soon after blood coagulation. Plasma samples were collected into siliconized tubes containing sodium fluoride and shipped to a central laboratory (SRL, Tokyo, Japan) for blood measurements. Serum triglycerides (TG), total cholesterol (TC), low-density lipoprotein (LDL) cholesterol, and high-density lipoprotein (HDL) cholesterol were measured using enzymatic methods, which have been standardized by the Lipid Standardization Program of the US Centers for Disease Control and Prevention/Cholesterol Reference Method Laboratory Network (CDC/CRMLN).^26^ Blood glucose was measured using hexokinase UV methods, and hemoglobin A1c (HbA1c) was measured using latex agglutination inhibition assays with the standardized method of the Japan Diabetes Society (JDS). HbA1c values were converted into the National Glycohemoglobin Standardization Program (NGSP) value using the following formula: HbA1c (NGSP) (%) = 1.02 × HbA1c (JDS) (%) + 0.25.^27^ Serum creatinine was measured enzymatically. High-sensitivity C-reactive protein (CRP) was measured using nephelometry methods, and brain natriuretic peptide (BNP) was measured via CLEIA using MI02 Shionogi BNP (Shionogi Co. Ltd., Osaka, Japan). Information on blood chemistry data measurements and their performance was described elsewhere.^26^

Spot urine samples were also collected and shipped to the same laboratory at which blood measurements were examined. Urine creatinine was measured enzymatically. Urine sodium and potassium were measured using selective ion electrode methods. Urine albumin and protein were measured using immuno-nephelometry and pyrogallol red methods.

### Follow-up

As the first step, the incidence of stroke, heart disease, and diabetes mellitus is surveyed annually from participants using the self-administered questionnaire via mail or telephone interviews. In the second step, when the incidence of these diseases is reported by participants or their family members, detailed medical records will be referred to the hospitals from the central office of the NIPPON DATA Research Group. The incidence of these diseases will then be assessed at the event adjudication committee of the study group. Information on medication for hypertension, dyslipidemia, and diabetes mellitus is also collected annually. The ADL and IADL survey is also performed every 5 years using the self-administered questionnaire.

Participants who die during the follow-up are confirmed by computer matching with the National Vital Statistics database, using area, sex, date of birth, and date of death as key codes, with the permission of the Management and Coordination Agency of the Government of Japan. The underlying causes of death in the National Vital Statistics database are coded according to the 10^th^ International Classification of Disease (ICD-10). Details of these classifications have been described elsewhere.^28^

### Main outcome measures

The study main outcome measures are listed in Table 2. All diagnoses will be based on medical records obtained from the hospitals. Each case is independently diagnosed by two trained medical doctors. When their diagnoses are mismatched, the committee adjudicates. The diagnostic criteria of main outcome events are described as follows.

**Table 2.** Main outcome measures: NIPPON DATA2010

Incidence	Stroke
	Ischemic stroke
	Hemorrhagic stroke
	Subarachnoid hemorrhage
	Unclassified
	Myocardial infarction
	Invasive treatment for coronary heart disease
	Arrhythmia
	Atrial fibrillation
	Sick sinus syndrome
	Atrioventricular block
	others
	Heart failure
	Diabetes mellitus
Medication	Hypertension
	Dyslipidemia
	Diabetes mellitus
Activities of daily living (ADL)	
Instrumental activities of daily living (IADL)	

### Heart disease

Myocardial infarction is diagnosed using the modified MONICA criteria or third universal definition of myocardial infarction by ESC/ACCF/AHA/WHF.^29^^,^^30^ Invasive procedures for coronary arteries, such as coronary artery bypass grafting (CABG) and percutaneous coronary intervention (PCI), are also considered as outcome events. Heart failure is diagnosed according to the Framingham Heart Study criteria.^31^ Researchers diagnose the case, taking into account symptoms and laboratory tests when the components of criteria are not fully collected. The acute exacerbation of chronic heart failure is also considered to be an outcome event when the case meets the Framingham criteria and requires hospitalization. Regarding arrhythmia, atrial fibrillation is diagnosed based on ECG findings. Sick sinus syndrome, atrioventricular block, and other arrhythmias are considered to be outcome events when the participant requires the insertion of a cardiac pacemaker.

### Stroke

Stroke is diagnosed with neurological symptoms that continue for 24 hours or longer.^32^ Secondary stroke due to injuries, leukemia, and tumors is excluded from outcome events. The stroke subtype is diagnosed based on imaging findings.

### Diabetes mellitus

Diabetes mellitus is diagnosed according to the modified criteria of the JDS as follows: 1) fasting blood glucose 126 mg/dL or higher, 2) casual blood glucose 200 mg/dL or higher, 3) HbA1c 6.5% or higher, and/or 4) medication for diabetes mellitus.^33^ A case that meets any one of these criterion items is considered to be an incident case of diabetes mellitus.

### Baseline descriptive statistics

The baseline characteristics of participants are shown in Table 3.

**Table 3A.** Physical characteristics of participants: NIPPON DATA2010 baseline (1,236 men and 1,655 women)

	Men	Women	*P* value
Number	1,236	1,655	
Age, years	60.0 (15.6)	58.0 (16.1)	0.001
BMI, kg/m^2^	23.9 (3.2)	22.7 (3.6)	<0.001
Systolic blood pressure, mm Hg	136.4 (18.0)	130.0 (20.0)	<0.001
Diastolic blood pressure, mm Hg	82.1 (10.8)	77.2 (10.8)	<0.001
Total cholesterol, mg/dL	201.5 (34.2)	208.7 (36.3)	<0.001
LDL cholesterol, mg/dL	118 (30.0)	119 (32.2)	0.208
HDL cholesterol, mg/dL	57 (14.9)	66 (15.1)	<0.001
Triglycerides, mg/dL	128 (90–190)	98 (67–142)	<0.001
HbA1c (NGSP), %	5.9 (0.9)	5.8 (0.7)	0.002
Co-morbidity, %			
Hypertension	58.2	42.5	<0.001
Diabetes mellitus	15.1	9.1	<0.001
Hypercholesterolemia	32.7	37.9	0.004
Hypertriglyceridemia	41.9	24.7	<0.001
Low HDL cholesterolemia	11.6	1.8	<0.001
Dyslipidemia	59.1	48.9	<0.001
Stroke	5.3	2.8	0.001
Myocardial infarction	3.2	0.7	<0.001
Medication, %			
Hypertension	31.7	24.6	<0.001
Diabetes mellitus	8.7	5.2	<0.001
Hypercholesterolemia	12.0	15.3	0.011
Hypertriglyceridemia	5.2	2.2	<0.001
Physical activity index	34.5 (30.4–40.5)	36.5 (32.9–40.3)	<0.001
Smoking, %			
Never	35.0	87.8	<0.001
Past	37.6	5.9	
Current	27.4	6.3	
Drinking, %			
Never	25.5	63.0	<0.001
Past	3.1	1.4	
Current	71.4	35.6	

BMI, body mass index; SBP, systolic blood pressure; DBP, diastolic blood pressure; LDL, low-density lipoprotein; HDL, high-density lipoprotein; TG, triglycerides; HbA1c, hemoglobin A1c.

Data were expressed as mean (standard deviation [SD]) for continuous variables, except triglycerides and the physical activity index (median [25%–75%]), and in % for dichotomous variables. *P* values for differences between men and women were estimated by *t*-tests for continuous variables, Mann Whitney tests for triglycerides and the physical activity index, and chi-squared tests for dichotomous variables.

Co-morbidities were defined as follows: Hypertension (SBP 140 mm Hg and higher and/or DBP 90 mm Hg and higher and/or on medication), diabetes mellitus (fasting blood glucose 126 mg/dL and higher and/or non-fasting blood glucose 200 mg/dL and higher and/or HbA1c 6.5% and higher and/or on medication. Samples obtained more than 8 hours after the last meal were considered to be fasting blood samples), hypercholesterolemia (LDL 140 mg/dL and higher and/or medication), hypertriglyceridemia (TG 150 mg/dL and higher and/or on medication), low HDL cholesterolemia (HDL-cholesterol less than 40 mg/dL), dyslipidemia (hypercholesterolemia and/or hypertriglyceridemia and/or low HDL cholesterolemia). A history of stroke and myocardial infarction was based on self-reports.

**Table 3B.** Socioeconomic characteristics of participants: NIPPON DATA2010 baseline

	Men	Women	*P* values
Number	1,236	1,655	
Age, years	60.0 (15.6)	58.0 (16.1)	0.001
Length of education, %			
>13 years	32.3	30.3	0.145
10–12 years	41.9	45.6	
<10 years	25.8	24.1	
Marital Status, %			
Married	81.1	72.7	<0.001
Single	18.9	27.3	
Living Status, %			
Living with others	91	87	0.004
Living alone	9	13	
Working status, %			
Unemployed	37.0	56.3	<0.001
Employed	63.0	43.7	
Self-employed	29.8	23.1	<0.001
Indefinite-term employee	60.4	55.9	
Limited-term employee	7.5	17.7	
Others	2.3	3.2	
Health Insurance, %			
National health insurance	40.9	38.0	0.088
Employees’ health insurance	41.2	45.1	
Medical care system for the elderly in the later stages of life	16.1	15.2	
Others	1.2	0.7	
Unknown	0.6	1.0	
Houseowner, %	81.4	81.4	0.998
Equivalent household expenditure, 10^4^ JPY	12.7 (8.9–17.5)	13.3 (9.0–17.5)	0.503
Household income, JPY, %			
<2,000,000	18.0	21.0	0.101
2,000,000–6,000,000	58.2	54.3	
>6,000,000	19.9	19.8	
Unknown	3.9	4.9	

JPY, Japanese Yen.

Data were expressed as mean (standard deviation [SD]) for age, as a median (25%–75%) for equivalent household expenditure (EHE), and in % for dichotomous variables. *P* values for differences between men and women were estimated by *t*-tests for continuous variables, Mann Whitney tests for EHE, and chi-squared tests for dichotomous variables.

Working status was categorized as follows: unemployed (unemployed, full-time homekeeper or retiree), self-employed (self-employed or family employee), indefinite-term employee (exective, indefinite-term employee or limited-term employee with 1 year and longer), limited-term employee (limited-term employee shorter than 1year).

EHE was estimated using the following formula: EHE = monthly household expenditure/square root of the number of household members.

### Strengths and limitations

We established NIPPON DATA2010 as a cohort study on a representative general Japanese population. Because the extraction method of study participants and ECG coding method were the same as in the NSCD, which were performed recently in 2000, we could consider NIPPON DATA2010 as the successor survey of NSCD. The study obtained not only physical measures, but also lifestyle factors, diet (NHNS2010), and socioeconomic factors (CSLC2010), and data collection and measurement methods were highly standardized.^26^ Thus, we will provide important information from multiple aspects for future strategies for CVD prevention and management in Japan. The integration method we adopted to use data from CSLC2010 may be applicable to NIPPON DATA80 and NIPPON DATA90, which will enable us to investigate the effects of changes in SES on health inequality from 1980.

Several limitations need to be noted for this study. The reporting bias for the measures obtained using the self-administered questionnaire, including data from NHNS and CSLC, may remain. Furthermore, the low response rates of NHNS may decrease the representativeness of this study. Nishi et al reported that the individual response rates of NHNS’s blood tests between 2003 and 2007 were between 31.6% and 39.3% via linking of CSLC and NHNS.^34^^,^^35^ They also showed that socio-demographic factors and lifestyle, such as being active, were related to cooperation for blood testing in NHNS. However, due to the lack of other cohort studies based on recent national surveys using a cluster random sampling design in Japan, NIPPON DATA2010 is considered to be the best available cohort that represents a recent Japanese population from all over Japan.

## APPENDIX A.

### NIPPON DATA2010 Research Group

Chairpersons: Katsuyuki Miura (Center for Epidemiologic Research in Asia and Department of Public Health, Shiga University of Medical Science, Otsu, Shiga, Japan) and Akira Okayama (Research Institute of Strategy for Prevention, Chuo, Tokyo, Japan).

Research members: Hirotsugu Ueshima (Center for Epidemiologic Research in Asia and Department of Public Health, Shiga University of Medical Science, Otsu, Shiga, Japan), Shigeyuki Saitoh (School of Health Sciences, Sapporo Medical University, Sapporo, Hokkaido, Japan), Kiyomi Sakata (Department of Hygiene and Preventive Medicine, Iwate Medical University, Yahaba, Iwate, Japan), Atsushi Hozawa (Department of Preventive Medicine and Epidemiology, Tohoku Medical Megabank Organization, Tohoku University, Sendai, Miyagi, Japan), Hiroshi Yanagawa (Jichi Medical University, Shimotsuke, Tochigi, Japan), Yosikazu Nakamura (Department of Public Health, Jichi Medical University, Shimotsuke, Tochigi, Japan), Tomonori Okamura (Department of Preventive Medicine and Public Health, Keio University School of Medicine, Shinjuku, Tokyo, Japan), Nobuo Nishi (International Center for Nutrition and Information, National Institute of Health and Nutrition, National Institutes of Biomedical Innovation, Health and Nutrition, Shinjuku, Tokyo, Japan), Nagako Okuda (Department of Health and Nutrition, University of Human Arts and Sciences, Saitama, Saitama, Japan), Takayoshi Ohkubo (Department of Hygiene and Public Health, Teikyo University School of Medicine, Itabashi, Tokyo, Japan), Fumiyoshi Kasagi (Institute of Radiation Epidemiology, Radiation Effects Association, Chiyoda, Tokyo, Japan), Yoshitaka Murakami (Department of Medical Statistics, School of Medicine, Toho University, Ota, Tokyo, Japan), Tohru Izumi (Kitasato University, Sagamihara, Kanagawa, Japan), Yasuhiro Matsumura (Faculty of Health and Nutrition, Bunkyo University, Chigasaki, Kanagawa, Japan), Toshiyuki Ojima (Department of Community Health and Preventive Medicine, Hamamatsu University School of Medicine, Hamamatsu, Shizuoka, Japan), Shinkan Tokudome (Nagoya City University Graduate School of Medical Sciences, Nagoya, Aichi, Japan), Hideaki Toyoshima (Nagoya University, Nagoya, Aichi, Japan), Hideaki Nakagawa (Medical Research Institute, Kanazawa Medical University, Uchinada, Ishikawa, Japan), Yoshikuni Kita (Faculty of Nursing Science, Tsuruga Nursing University, Tsuruga, Fukui, Japan), Aya Kadota (Center for Epidemiologic Research in Asia and Department of Public Health, Shiga University of Medical Science, Otsu, Shiga, Japan), Akira Fujiyoshi, Naoyuki Takashima (Department of Public Health, Shiga University of Medical Science, Otsu, Shiga, Japan), Naomi Miyamatsu (Department of Clinical Nursing, Shiga University of Medical Science, Otsu, Shiga, Japan), Yasuyuki Nakamura (Department of Food Science and Human Nutrition, Ryukoku University, Otsu, Shiga, Japan), Takehito Hayakawa (Research Center for Social Studies of Health and Community, Ritsumeikan University, Kyoto, Kyoto, Japan), Katsushi Yoshita (Department of Food and Human Health Science, Osaka City University Graduate School of Human Life Science, Osaka, Osaka, Japan), Yoshihiro Miyamoto (Department of Preventive Cardiology, National Cerebral and Cardiovascular Center, Suita, Osaka, Japan), Kazunori Kodama (Radiation Effects Research Foundation, Hiroshima, Hiroshima, Japan), Yutaka Kiyohara (Hisayama Research Institute for Lifestyle Diseases, Hisayama, Fukuoka, Japan), Hisatomi Arima (Department of Preventive Medicine and Public Health, Faculty of Medicine, Fukuoka University, Fukuoka, Fukuoka, Japan), Toshiharu Ninomiya (Department of Epidemiology and Public Health, Graduate School of Medical Sciences, Kyushu University, Fukuoka, Fukuoka, Japan) and Kazuo Ueda (Murakami Memorial Hospital, Nakatsu, Oita, Japan).

### Japanese public health centers which cooperated with NIPPON DATA2010’s baseline survey

Chitose Health Care Center, Takikawa Health Care Center, Yakumo Health Care Center, Obihiro Health Care Center, Abashiri Health Care Center, Kitami Public Health Center, Sapporo City Public Health Office, Asahikawa Health Center, (Hokkaido), Goshogawara Public Health Center, Aomori City Health Center, (Aomori), Ken’o Public Health Center, Ofunato Health Center, Ninohe City General Welfare Center, (Iwate), Shiogama Health Center, Osaki Health Center, Sennan Health Center, Aoba Branch, Sendai Public Health Center, Miyagino Branch, Sendai Public Health Center, (Miyagi), Daisen Public Health Center, Akita City Public Health Center, (Akita), Murayama Health Center, Okitama Health Center, (Yamagata), Ken-poku Public Health and Welfare Office, Ken-chu Public Health and Welfare Office, Ken-nan Public Health and Welfare Office, Soso Public Health and Welfare Office, (Fukushima), Mito Public Health Center, Hitachiomiya Public Health Center, Chikusei Public Health Center, Joso Public Health Center, (Ibaraki), Eastern Tochigi Prefectural Health Center, Ansoku Public Health And Welfare Center, Utsunomiya City Public Health Center, (Tochigi), Tonenumata Health and Welfare Office, Seibu Health Center, Kiryu Health and Welfare Office, (Gunma), Kawaguchi Public Health Center, Asaka Public Health Center, Chigasaki Public Health and Welfare Center, Sayama Public Health Center, Kasukabe Public Health Center, Soka Public Health Center, Satte Public Health Center, Saitama City Public Health Center, Kawagoe City Public Health Center, (Saitama), Ichikawa Health and Welfare Center, Matsudo Health and Welfare Center, Noda Health and Welfare Center, Inba Health and Welfare Center, Kimitsu Health and Welfare Center, Kaisou Health and Welfare Center, Chiba City Health Center, Funabashi Public Health Center, Kashiwa City Health Center, (Chiba), Tama Fuchu Public Health Center, Nishitama Public Health Center, Minamitama Public Health Center, Tama Tachikawa Public Health Center, Tama-Kodaira Public Health Center, Bunkyo Public Health Center, Sumida City Public Health Center, Koto City Public Health Center, Shinagawa City Public Health Center Ebara Health Center, Ota City Public Health Center Chofu Area Health Division, Ota City Public Health Center Kojiya and Haneda Area Health Division, Setagaya Public Health Center, Shibuya City Public Health Center, Suginami Public Health Center, Kita City Public Health Center, Itabashi City Public Health Center, Nerima City Public Health Center, Takenotsuka Public Health Center, Katsushika City Public Health Center, Shishibone Public Health Support Center, Hachioji-shi Health Center, (Tokyo), Kamakura Public Health and Welfare Center, Atsugi Public Health & Welfare Center, Kanagawa Ward Health and Welfare Center, Nishi Ward Health and Welfare Center, Naka Ward Health and Welfare Center, Kanazawa Ward Health and Welfare Center, Totsuka Ward Health and Welfare Center, Konan Ward Health and Welfare Center, Midori Ward Health and Welfare Center, Tsuzuki Ward Health and Welfare Center, Saiwai Public Health Centers, Nakahara Public Health Centers, Takatsu Public Health Centers, Sagamihara Public Health Center, Yokosuka City Health Center, Fujisawa City Health Center, (Kanagawa), Sanjo Public Health Center, Tokamachi Public Health Center, Sado Public Health Center, Niigata City Health Center, (Niigata), Takaoka Health Center, Toyama Municipal Health Center, (Toyama), Minami-Kaga Public Health Center, Kanazawa Healthcare Center, (Ishikawa), Fukui Health and Welfare Center (Fukui), Kyotoh Public Health Branch, Fuji-Tobu Public Health Branch, (Yamanashi), Saku Public Health Center, Ina Health and Welfare Office, Matsumoto Health Center, Nagano Health Center, (Nagano), Tono Public Health Center, Seino Public Health Center, Kamo Public Health Center, Gifu City Public Health Center, (Gifu), Gotemba Public Health Center, Fuji Public Health Center, Chubu Public Health Center, Seibu Public Health Center, Shizuoka City Public Health Center, Hamamatsu Nishi Ward Office, Hamamatsu Higashi Ward Office, (Shizuoka), Ichinomiya Health Center, Seto Health Center, Handa Health Center, Kasugai Health Center, Toyokawa Health Center, Nishio Health Center, Kinuura-Tobu Health Center, Naka Health Center, Showa Health Center, Minato Health Center, Midori Health Center, Meito Health Center, Tempaku Health Center, Toyohashi Public Health Center, Okazaki City Public Health Center, (Aichi), Kuwana Public Health Center, Suzuka Public Health Center, Tsu Public Health Center, Ise Public Health Center, (Mie), Kusatsu Public Health Center, Koka Public Health Center, Otsu City Public Health Center, (Shiga), Otokuni Public Health Center, Tango Public Health Center, Nakagyo Public Health Center, Ukyo Public Health Center, Nishikyo Public Health Center, (Kyoto), Ikeda Public Health Center, Toyonaka Public Health Center, Suita Public Health Center, Ibaraki Public Health Center, Hirakata City Public Health Center, Fujiidera Public Health Center, Tondabayashi Public Health Center, Kishiwada Public Health Center, Izumisano Public Health Center, Higashisumiyoshi Ward Public Health and Welfare Center, Nishinari Ward Public Health and Welfare Center, Nishiyodogawa Ward Public Health and Welfare Center, Osaka City Public Health Office, Higashiyodogawa Ward Public Health and Welfare Center, Hirano Ward Public Health and Welfare Center, Kita Ward Public Health and Welfare Center, Sakai City Sakai Public Health Center, Higashiosaka City Public Health Center, (Osaka), Itami Health & Welfare Office, Akashi Health & Welfare Office, Kato Health & Welfare Office, Sumoto Health & Welfare Office, Kobe City Public Health Center, Amagasaki City Health Center, Nishinomiya City Health Center, (Hyogo), Koriyama Public Health Center, Nara-city Public Healthcare Center, (Nara), Tanabe Public Health Center, Wakayama City Health Center, (Wakayama), Kurayoshi Public Health Center, (Tottori), Hamada Public Health Center, Masuda Public Health Center, (Shimane), Mimasaka Health Center, Mimasaka Health Center Shoei Branch, Public Health Center, City of Okayama, Kurashiki Public Health Center, (Okayama), Western Center for Public Health, Naka Public Health Center, Hiroshima City, Minami Public Health Center, Hiroshima City, Asakita Public Health Center, Hiroshima City, Fukuyama City Health Center, (Hiroshima), Iwakuni Health & Welfare Center, Yanai Health & Welfare Center, Shimonoseki City Health Center, (Yamaguchi), Tokushima Public Health Center, (Tokushima), Tosan Regional Health and Welfare Office, Chusan Regional Health and Welfare Office, (Kagawa), Yawatahama Burreau Department of Health, Welfare and Environment Matsuyama City Health Center, (Ehime), Aki Public Health and Welfare Office, Kochi City Health Center, (Kochi), Munakata·Onga Office for Health, Human Services and Environmental Issues, Tagawa Office for Health and Human Services, Kaho·Kurate Office for Health, Human Services and Environmental Issues, Kasuya Office for Health and Human Services, Itoshima Office for Health and Human Services, Public Health Service and Assistance Section, Tobata Ward Office, Public Health Service and Assistance Section, Kokurakita Ward Office, Hakata Ward Public Health Center, Minami Ward Public Health Center, Sawara Ward Public Health Center, Higashi Ward Public Health Center, (Fukuoka), Kito Public Health and Welfare Office, (Saga), Ken’ou Healthcare Office, Nagasaki City Public Health Center, (Nagasaki), Kikuchi Public Health Office, Amakusa Public Health Office, North Health and Welfare Center, Health and Welfare Section Ueki General Branch, (Kumamoto), Northen Public Health Center, Oita Municipal Public Health Center, (Oita), Miyakonojo Public Health Center, Nobeoka Public Health Center, Miyazaki Municipal Health Center, (Miyazaki), Aira Public Health Center, Tokunoshima Public Health Center, Kagoshima City Health Center, (Kagoshima), Miyako Regional Welfare and Public Health Center, Chūbu Regional Welfare and Public Health Center, (Okinawa).

### Medical examination institutions which cooperated with NIPPON DATA2010’s baseline survey

The Center for Comprehensive Health Check and Promotion, Japan Anti-Tuberculosis Association (Tokyo), Hokkaido Anti-Tuberculosis Association, Sapporo Clinical Laboratory Inc. (Hokkaido), Aomori General Health Examination Center (Aomori), Iwate Health Service Association (Iwate), Miyagi Anti-Tuberculosis Association (Miyagi), Akita Foundation For Health Care (Akita), Public Interest Foundation of Yamagata Health Promotion System (Yamagata), Fukushima Preservative Service Association for Health (Fukushima), Ibaraki Health Service Association Institute (Ibaraki), Tochigi Public Health Service Association (Tochigi), Gunma Health Foundation (Gunma), Saitama Health Promotion (Saitama), Chiba Foundation for Health Promotion & Disease Prevention (Chiba), Tokyo Anti-Tuberculosis Association (Tokyo), Kanagawa Anti-Tuberculosis Association, Kanagawa Health Service Association (Kanagawa), Niigata Health Service Center (Niigata), Toyama Prefecture Health Promotion Center (Toyama), Center for Disease Prevention and Health Promotion Ishikawa (Ishikawa), Fukui Health Service Association (Fukui), Yamanashi Health Management Association (Yamanashi), Nagano health promotion corporation (Nagano), Gifu General Health Checkup Center, Gifu Health Care Medical Center (Gifu), Foundation The Japan Anti-Tuberculosis Association Shizuoka Branch (Shizuoka), Aichi Health Promotion Public Interest Foundation, Public Health Association. Inc., Handa Medical Association Health Care Center, Toyohashi Medical Association, Kariya Medical Association Clinical Laboratory Center, The Aichi Clinic of Healthcare, Chukyo Satellite Clinic, Chubu Medical Practice Sanitary Survey Center Chubu Clinic (Aichi), Mie Healthcare Center (Mie), Shiga Health Promotion Foundation (Shiga), Kyoto Preventive Medical Center (Kyoto), Osaka Anti-Tuberculosis Association (Osaka), Hyogo Prefecture Health Promotion Association, Hyogo Health Service Association, Amagasaki Health Medical Foundation (Hyogo), The Nara Foundation of Longevity (Nara), Wakayama Medical Examination Center (Wakayama), Tottori Health Service Association (Tottori), Shimane Environment & Health Public Corporation (Shimane), Okayama Health Foundation (Okayama), Foundation for Community Health and Medicine Promotion in Hiroshima Prefecture (Hiroshima), Yamaguchi Health & Service Association (Yamaguchi), Tokushima Health Screening Center (Tokushima), Kagawa Health Service Association (Kagawa), Ehime General Health Care Association (Ehime), Kochi Health Service Association (Kochi), Fukuoka Anti-Tuberculosis Association (Fukuoka), Saga Health Promotion Foundation (Saga), Nagasaki Prefecture Medical Health Operation Group (Nagasaki), Kumamoto General Health Center (Kumamoto), Public Interest Foundation Oita Prefecture Regional Health Support Center (Oita), Miyazaki Prefectural Health Foundation (Miyazaki), Kagoshima Prefectural Comprehensive Health Center (Kagoshima), Okinawa Health Promotion Foundation (Okinawa).
